# Supplementary material for: Nursing Students’ Perceptions of Clinical Debriefing TALK©: A Qualitative Case Study
Source: Nurs Rep. 2025 May 30;15(6):194. doi: 10.3390/nursrep15060194 (PMC12196150; doi:10.3390/nursrep15060194)
Supplement: Supplementary file 1 [file nursrep-15-00194-s001.zip › nursrep-3556864-supplementary.pdf]

Narratives	Code	Category	Subtheme	Theme
P19: if you experience a situation and it is a situation that impacts you in some way, good or bad, innately you are already going to reflect on that situation [...]. Now, doing a debriefing dedicated to that specifically is going to help you more than doing it by yourself in a natural way, it is giving you an objective, which is to look for points of improvement and to analyze the situation.	Structured reflection for improvement	1. Reflexivity in clinical debriefing	1.1 Reflexivity.	**1. Process and Structure of Clinical Debriefing.**
P26: if you are the one who decides it is because you want to draw conclusions from these things or solutions and learn, or in your head it is because you kept thinking about it, so there was something that you wanted to solve [...]. I think you are more open to reflect.	Voluntary reflection and problem-solving drive	2. Learning objectives and reflexivity		
P17: For me, it was a moment that provided space to express if you have any other concerns, and to know that you can count on others who are in the same situation as you. And to be able to solve that problem from different perspectives. And in that way, you can find a practical solution.	Peer support and shared problem-solving	3. Multiple perspectives		
P13: You start developing more critical thinking when you become aware of the mistakes you make. When you continue doing clinical practice, you stop and ask yourself… why am I doing this, and why not something better? You train your mind not to do things automatically.	Development of critical thinking and self-regulation	4. Autopilot mode and critical thinking		

P26: With limited knowledge, you can't engage in deeper reflection. Now, in my fourth year and with more knowledge, I can say, for example, the doctor did this for that reason. You feel more motivated to reflect on.	Knowledge depth enhances reflective capacity	5. Knowledge		
P6: In a debriefing, it's not the person who knows the most who should speak the most—especially when the goal is to learn how to manage patient care effectively in an interdisciplinary team. Everyone needs to participate equally, regardless of their level of knowledge, in order to provide quality care.	Equity and inclusivity in interdisciplinary reflection			
P20: We discussed a case in the operating room. There was a laparoscopic digestive surgery, and they ended up sectioning the hepatic artery, and then there was massive bleeding and analyzed what we thought we could have done or what could have been done to avoid it or how the thing flowed when it happened.	Learning from real clinical errors	6. Error management		
P20: We discussed a case in the operating room. There was a laparoscopic digestive surgery, and they ended up sectioning the hepatic artery, and then there was massive bleeding and analyzed what we thought we could have done or what could have been done to avoid it or how the thing flowed when it happened.	Student reflection on clinical decision-making and error prevention	7. What students reflect upon		
P6: For sure what touches you up close is engraved in your heart, isn't it? That is so. The others will also learn something, but it won't be so engraved, it won't remind you of that pa-tient, it may remind you of what we talked about in the debriefing.	The protagonist emotional imprint and memory in reflection	8. The protagonist		

P6: It is a good way to get to the end of the debriefing, which is to look for that solution. It allows you to have a much more holistic, much more general view of the case, and I think that also allows you to analyze it better, reflect better and draw better conclusions.	Holistic analysis and deeper reflection through debriefing because of the facilitator	9. The facilitator using the TALK© tool	1.2 Approach and Technique.	
P19: I think it has to be someone external, neutral, and who handles the tool well for it to be effective.	Need for neutrality and facilitator competence	10. The neutral facilitator		
P5: It seems to me that, if we do not choose the topics to talk about and one is imposed on us, maybe we are not so willing to comment on that case or maybe we have not experienced anything similar. [...] Well, you don't get so much into it and you won't get so much out of it.	Participant agency enhances engagement	11. Student involvement		
P10: I think they were guiding us with the TALK tool, because otherwise... we would've gone off track. But it wasn't super rigid—like, if we were on the 'L' step, we weren't forbidden from going back.	Flexible guidance using TALK tool	12. Flexibility of the TALK© tool		
P8: Sometimes you don't bring up the topic yourself… for whatever reason, and if the facilitators know what happened and that it's useful to talk about it, it might be important.	Facilitator intervention in sensitive and important topics	13. When it is important to talk about something		
P22: They were questions to make you think about why, and not to judge you or question you, but to reflect.	Reflective questioning without judgment	14. Facilitators' questions		

P5: You are with them every afternoon, we get together to have a snack and I think you form a special bond with these people while you are doing internships with them. So, in the end, we have that feeling, we are comfortable there. [...] As I already had the previous experience of the ICU... better, you get to know the tool better and you follow it more, you are more comfortable.	Familiarity and bonding enhance comfort	15. Experience and familiarity		
P6: Not in the heat of the moment. I don't like to talk about things in the heat of the moment. Especially if it has to be something negative. I think that after... after a week or so, or even a few days, that's when it's better. [...] I mean, out of the storm everything is much clearer.	Temporal distance improves reflective clarity	16. Timing of clinical debriefing	1.3 Moment and Context.	
P7: the sooner you talk about it, the sooner other people can also realize that I can also make a mistake in that and you can also prevent other people's mistakes.	Early discussion helps prevent future errors			
P21: there are differences because, well, each service is different. So, well, debriefing? I think that the dynamics of debriefing would not really change. It would simply change because of the content or errors that may occur, which may be more serious.	Debriefing varies with context and service	17. Clinical debriefing in each department		
P19: In the end, I think that the ICU is an internship where students have a lot of relationship with the rest of the classmates, so you develop more confidence. In other interships where you may not have as much connection, debriefing can be difficult, as in the operating room.	Peer relationships influence debriefing dynamics			

P5: But the fact that it is a first time is like you have never experienced anything like it before. So that marks you, you are not used to it, and it impacts you. And you take that with you. And it's important, it's like you learn more from it.	Impact of first-time clinical experiences	18. First-time experiences		
P6: Clinical debriefing has a real objective for the well-being of the patient and to get something out of it. What we tell at snacks is often not a reflective process, it is an absolute avoidance process.	Contrast between structured and informal reflection	19. Conversations over coffee breaks		
P21: Let's see, it could be done in the second... in the third and fourth year to see the evolution. I think it would never hurt. But now that's it, you're on the edge, you're on the edge of student life and what I've learned in it, and the abyss of... "hello, let's see what comes to me."	Reflection at the transition to professional practice	20. Debriefing across different stages of training		
P5: I feel that learning is more visible, that is, you assimilate it more if you relate it to a feeling that it has produced in you, it is more marked.	Emotion-linked learning is more memorable	21. Connection to emotional experience	2.1 The Emotion of The Experience.	**2. The Emotional Sphere.**
P7: if something goes wrong in a simulation it doesn't affect me, because I say, well, I did it wrong, but nobody got hurt. [...] In the clinical debriefing, if we comment on the fact that I have inadvertently made a medication error, then I know that I will get more involved.	Real consequences heighten involvement	22. Reality		
P6: I felt a bit like saying, it wasn't that hard either [...] So, on the one hand, learning, and on the other hand, it was like, you could have done better. Like feeling, it's not like feeling dumb, but a little bit of guilt.	Mild guilt as a trigger for learning	23. Judgment and guilt in clinical debriefing	2.2 Emotional Management and Support.	

P19: Yes, there was some issue that could... could lead to that because we talked about medication errors and things like that. But other than that burden of guilt that already comes with the issue innately, no, I didn't feel judged or anything like that.	No judgment despite sensitive topics			
P8: I think that with the group I had it was minimized, that is, if P6 could feel guilty, then the rest of the colleagues made him feel that he was not guilty.	Group containment reduces guilt			
P15: It is very hard to open up and say what we feel, and thanks to this you can say, well, look, I was so scared that I ran away. It makes it easier to talk. You don't always have the opportunity or the space to express your feelings.	Debriefing enables emotional expression	24. Impact, support, and emotional management		
P10: Well, a little bit of what my classmates have said about, well that, by talking about it and so on, knowing that it could not only have happened to you, and also feeling supported and listened to a little bit.	Shared experience fosters empathy and support			
